# The Five Diaphragms in Osteopathic Manipulative Medicine: Neurological Relationships, Part 2

**DOI:** 10.7759/cureus.8713

**Published:** 2020-06-20

**Authors:** Bruno Bordoni

**Affiliations:** 1 Physical Medicine and Rehabilitation, Foundation Don Carlo Gnocchi, Milan, ITA

**Keywords:** diaphragm, osteopathic, fascia, myofascial, fascintegrity, physiotherapy

## Abstract

The main objective of the osteopath and that of osteopathic manipulative medicine (OMM) is to create space between the different tissues. The sliding capacity of the various tissue layers and between the different body components, up to the possibility of movement between cells is the salutogenic stimulus to allow the circulation of fluids, the biochemical exchange, and the adequate management of the multiple internal and external stimuli that perturb the body living. Movement is allowed by space and space is life. In this second part, the exposure of the anatomical neurological relationships of the five diaphragms continues, highlighting the relationships of the thoracic outlet, the respiratory diaphragm, and the pelvic floor. Finally, there will be clinical reflections to further corroborate the existence of the anatomical continuum and to lay the scientific foundations for an OMM approach to body diaphragms.

## Introduction and background

In the first part, we discussed the neurological relationships of the tentorium cerebelli and the muscular complex of the tongue; the latter two constitute with the thoracic outlet, the respiratory diaphragm, and the pelvic floor what in osteopathic manipulative medicine (OMM) is called the model of the five diaphragms [1]. The five diaphragms reflect the concept of another historical model with which the clinician guides the evaluation and the clinical decision, that is, the respiratory-circulatory model. The philosophy behind the latter and the five diaphragms is to allow the creation of space between the different body tissues (muscular, visceral, vascular, and nervous), as if there is the possibility of a better movement of the tissues (and therefore space) it is possible to find greater health and well-being for the patient [2]. Breathing and blood/lymphatic circulation allow better salutogenic homeostasis; the movement of the tissues, the increased space between the tissues themselves and the free movement of fluids is a health status. In literature, no text contains the description of the neurological relationships between the five diaphragms, that is, those body segments that can be considered as diaphragms. From this need, the desire to write a two-part text describing these neurological connections arises. The possibility of adequate movement of tissues and blood, nervous and lymphatic vessels is vital for maintaining health. There is a close relationship between the path of the nervous system and the presence of blood and lymphatic vessels, as well as vasa nervorum. Inflammation of the latter system can induce a neuropathy (peripheral and cranial nerves) known as nonsystemic vasculitic neuropathy (NSVN), with the possible occurrence of a sensorimotor chronic neuropathic pain [3]. The vasa nervorum creates different anastomoses along with the entire thickness of the nerve (epinerium, perinerium, endonerium) and their inflammatory involvement will create damage from the skin to the muscles; the causes are not fully understood [3]. The vasa nervorum is innervated by filaments from the nerve that vascularize, that is, the nerves nervorum, with sympathetic and parasympathetic components [4]. The tissues penetrated by the different nerves are responsible for the stimuli that come from the nerve itself: mechanical stimuli (lengthening, shortening), compressions, thermal biochemical stimuli [5]. If a tissue affected and penetrated by the nerve path creates problems in the sliding of the nerve, the nervorum nerves will perceive this difficulty, turning into potential vehicles of nociceptive stimuli [5]. The arterial vessels that feed the tissues and the vasa nervorum can cause local and systemic pain and inflammation if their ability to sense mechanical and metabolic stimuli is altered; the vessels penetrate many tissues with very long paths and if these passages are limited by the tissues they encounter, the vessel will suffer [6]. The outermost layer of the vessel (adventitia) is rich in fibroblasts, leukocytes, microvessels, stem cells, and fat; if the vessel suffers, it will become more rigid with paracrine and autocrine reactions of inflammatory substances and with nociceptive stimulation of the nerve vasorum or nerva vasorum [6]. The ability of the nerve and vessel to slide along the tissues they encounter influences the general ability of musculoskeletal movement. The nerve and vessel tissue can undergo a nonphysiological alteration of the intrinsic and phenotypic properties, which give shape and function to the nervous and vascular structure. If this scenario occurs locally or in a systemic context, the same vessel/nerve will prevent the tissues that are penetrated (from the nerves/vessels) from moving correctly, altering the pattern of musculoskeletal movement [7]. To give an example, the movement of the ankle can be reduced due to a lower elasticity of the sciatic nerve during the movement of the foot (dorsiflexion) [7]. A less elastic nerve changes the orientation of the fibers of the basal lamina, decreases the hysteresis capacity, with plastic structural adaptations [8]. If the tissues are not free to slide over each other, a mechanometabolic dysfunction will be created, starting from densification of the tissues up to an alteration of the extracellular matrix [9]. If the extracellular matrix alters the mechanical environment, the change will induce a nonphysiological lymphatic vascular morphogenesis [10]. The nervous system and the lymphatic system are bi-directionally affected; a dysfunction of a nerve due to poor ability to move will negatively affect the lymphatic and lymph node response, which could trigger an inflammatory process on the nerve [11]. A treatment of diaphragms with OMM is able to improve the movement and health of the patient [12-14].

## Review

Thoracic outlet: cervical plexus and sympathetic cervical chain

**Figure 1:**
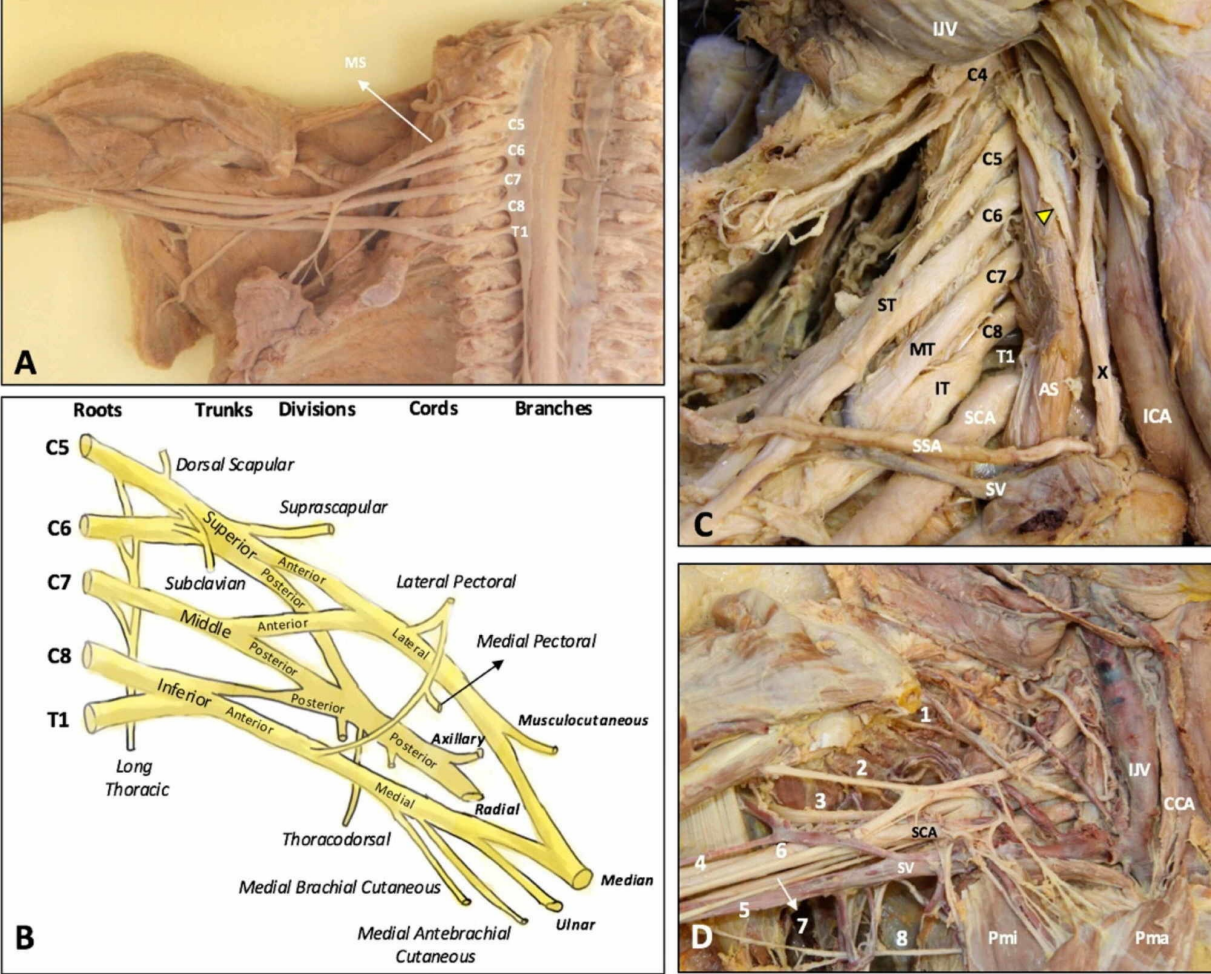
Overview of the brachial plexus (BP) on the cadaveric model after removal of the anterior scalene muscle (A). Schematic drawing of the BP anatomy (B). The roots, trunks, divisions, cords (C), and terminal branches (D) of the BP in the cadaveric models. 1: suprascapular nerve; 2: musculocutaneous nerve; 3: axillary nerve; 4: radial nerve; 5: medial brachial cutaneous nerve; 6: median nerve; 7: ulnar nerve; 8: intercostobrachial cutaneous nerve. AS: anterior scalene muscle; MS: middle scalene muscle; CCA: common carotid artery; ICA: internal carotid artery; IJV: internal jugular vein; SCA: subclavian artery; SV: subclavian vein; SSA: suprascapular artery; ST: superior trunk; MT: middle trunk; IT: inferior trunk; Pma: pectoralis major muscle; Pmi: pectoralis minor muscle; X: vagus nerve. Yellow arrowhead: phrenic nerve. Images reproduced with permission of Dr Po-Cheng Hsu and colleagues, Department of Physical Medicine and Rehabilitation, National Taiwan University Hospital, Bei-Hu Branch, Taipei, Taiwan.

The brachial plexus (BP) is located in the thoracic outlet area, identifiable from the lateral portion of the scalene muscle anterior to the lower portion of the pectoralis minor muscle [15]. BP is the neurological structure that allows somatosensory innervation of the upper limb and part of the thoracic outlet [16]. The ventral branches of the spinal nerves from C5 to T1 form BP; it is not uncommon for C4 and T2 roots to converge to immerse themselves in BP [15]. The anterior or ventral branches are the roots found in the neck in the scalene triangle (anterior and middle scalene muscle); the diameter of C5 is the smallest, compared to the volume of C7 and C8, which roots are the largest [15]. The branches will form the upper trunk (C5-C6), the middle trunk (C7), and the lower trunk (C8-T1); the trunks are formed after the scalene triangle near the subclavian artery [17]. The trunks continue towards the clavicle and once past the clavicle bone, the trunks are further divided into an anterior and posterior division, for a total of six divisions: the anterior division will be formed by the upper and middle trunk, while the posterior division it will be formed by the upper, middle, and lower trunk [16-17]. Generally, the anterior division will provide innervation to the flexor muscles of the arm, while the posterior division will provide innervation to the extensor muscles [16]. In the proximity of the first rib and the subclavian muscle, the divisions will create three cords. The lateral cord (central position, C5-C7) will come out of the upper and middle trunks; the posterior cord will form from the posterior trunk (cranial position, C5-T1); the lower trunk will form the middle cord (caudal position, C8-T1) [15-16]. In the lateral area of the pectoralis minor muscle and passing under the coracoid process of the scapular bone, the cords are further divided into five terminal branches at the axilla level. We can recognize the axillary nerve, the ulnar nerve, the median nerve, the musculocutaneous (or perforating Casserius) nerve, and the axillary nerve [16]. Before the nerves come out to form the ventral and dorsal roots, they form intradural anastomoses, throughout the spinal path excluding the sacral area; the area of C3-C6 is the cervical portion where these connections are most commonly found [18]. These connections with myelinated fibers are formed between dorsal rootlets and the spinal level below or above. There are many anastomoses between the BP cords and the path of the different terminal nerves and this highlights the absolute nervous continuum; in addition, branches of the cervical plexus involve the cutaneous area of the thoracic outlet such as the supraclavicular nerve [19]. We can find several nerves before branching into terminal branches. From C4 in some subjects, the levator nerve of the clavicle arises, in greater percentage from the left side; also from C4, the sensitive supraclavicular nerve with different branches that pass beyond the clavicle superficially arises, bringing the sensitivity of the clavicular area, the proximal portion of the chest, and the anteromedial area of the shoulder [20-21]. The root of C5 contributes to the formation of the dorsal scapular nerve, together with the roots of C3-C4 and the cranial nerve XI they form the motor pathway for the levator muscle of the scapula and the rhomboid muscles [22]. The suprascapular nerve derives from the upper trunk of the BP from C5-C6 and has motor functions for the supraspinatus and infraspinatus muscles, and with sensitive functions for the shoulder area (70% of the sensitivity of the shoulder) [15]. The subclavius nerve starts from C5-C6 (motor nerve); the subscapular nerves (C5-C6) derive from the posterior cords of the BP, with an upper and lower branch innervating the subscapular muscle and the teres major muscle [15]. The long thoracic nerve affects the roots of C5-C6 and sometimes C7, with contributions that can also come from C4 and C8; it innervates the anterior serratus muscle and runs parallel with the dorsal nerve of the scapula [15]. The pectoral nerves (C5-T1) are divided into lateral and medial; the medial nerves derive from the medial cord of the BP and run along the lower edge of the pectoralis minor innervating it and also giving innervation to the lower and middle portion of the pectoralis major. The lateral branches originate from the lateral cord and are directed with the thoracoacromial artery and the vein below the surface of the proximal portion of the pectoralis major to innervate it [15]. The lateral pectoral nerve can innervate the clavicular portion of the deltoid muscle. The pectoral nerves also have a sensitive component, in particular the lateral branches, innervating the acromioclavicular joint, the coracoclavicular ligaments, the subacromial bursa together with the branches of the suprascapular nerve, the periosteum of the clavicle. The lateral pectoral nerve anastomoses with the subclavian nerve [15]. The middle cord originates from the subscapularis nerve (C5-C6), with upper and lower branches (Figure 1) [15].

The phrenic nerve and the vagus nerve will be discussed later in the article. An important structure that we find in the cervical path and that involves the tentorium cerebelli, the tongue, and the thoracic outlet is the sympathetic system, like the sympathetic cervical chain, wrapped by the prevertebral fascia. The cervical sympathetic trunk (CST) consists of three ganglia: upper (level C2-C3), medium (level C6-C7), lower (stellate ganglion, C7-T1) [17]. The superior ganglion is the most voluminous in the lateral proximity of the longus colli muscle and the transverse process of the second and third cervical vertebrae, medial to the internal carotid artery; the middle ganglion is the smallest in volume and is in close proximity to the lower thyroid artery and lateral to the common carotid artery and the intervertebral disc between the C6 and C7 vertebra, with a percentage of presence of only 41%. The inferior ganglion is a fusion between the inferior ganglion of the CST and the first sympathetic thoracic ganglion; it has different shapes (star or stick) and has a medium volume compared to the first cervical ganglion and positioned near the first rib and the transverse process of C7, lateral to the longus colli muscle and posterior to the vertebral artery [17]. In the lower portion of the stellate ganglion (SG) or cervicothoracic ganglion we can recognize Sibson's pleural fascia, which covers the apex of the lung [23]. The CST communicates with all the cervical roots and can also consist of a fourth pair of ganglia near the vertebral arteries; SG can also merge with the second sympathetic thoracic ganglion up to the fourth thoracic ganglion [23]. From the SG arises multiple filaments such as the vertebral nerve. The vertebral nerve ascends to the root of C3, creating anastomosis with the latter and creating a sympathetic plexus on the vertebral artery; fibers of the vertebral nerve can innervate portions of prevertebral muscles, by a motor and sensory point of view [24]. Additional fibers innervate the intervertebral and zygapophyseal joints and more rarely meningeal fibers can be recognized; thinner fibers follow the superior cerebellar artery and the basilar artery, entering the skull [24]. Stimulation of this nerve can cause eye movement with pupillary changes, including symptoms such as dizziness and headaches [24]. SG sends post-ganglion branches in the lower direction, forming several sympathetic plexuses. We find a plexus on the subclavian artery, a cardiac branch will help form a cardiac plexus [23]. Other efferences, through animal studies, from the SG will go to different viscera (trachea, esophagus, heart, thyroid, stomach). In animal studies, afferences that go towards the SG from different body parts have been highlighted: from the motor nuclei of the vagus, from the spinal ganglia of C8 to T19, from the spinal cord of the C8-T10 tract, and from afferents of neurons inside the ganglia visceral intramural (esophagus, trachea, heart, aortic arch, lungs) [25]. Somatic fibers will derive from the gray branches communicating towards the spinal roots. The pericardiophrenic artery sends a branch to vascularize the SG. The white communicating branch connects the first thoracic nerve with the SG; the first and / or second intercostal nerve anastomizes with SG and BP, via the Kuntz nerve. The T2 nerve or intercostobrachial nerve anastomizes with the pectoral nerves and with the thoracodorsal nerve and with the supraclavicular nerve, consequently contacting the cervical plexus; it anastomizes with the lateral cutaneous antibrachial nerve and the posterior cutaneous antibrachial nerve, which last are the continuation of the musculocutaneous nerve and from the posterior cord of the BP entering the radial nerve, respectively [25]. We will later see other anastomoses with the SG with the phrenic nerve and the X. The area of the thoracic outlet or upper thoracic diaphragm must allow the passage of the BP and avoid functional disturbances to the SG. There are efferent connections of the SG with the trigeminal nucleus but we do not know well the functions of this connection. Animal studies show that post-ganglionic efferences involve the upper limb, up to the joints; probably, these connections could cause possible joint inflammations [26]. SG efferences come to influence the temporomandibular joint, modulating the pain of this joint [27]. SG is involved in pain syndromes (e.g. Ramsay Hunt Syndrome, Burning Mouth Syndrome, congenital venous malformations of the arm) and in other pathologies or disorders that cause pain (tissue deoxygenation) not always clinically framed, involving the skull, the cervical tract, and the tongue.

Respiratory diaphragm

**Figure 2:**
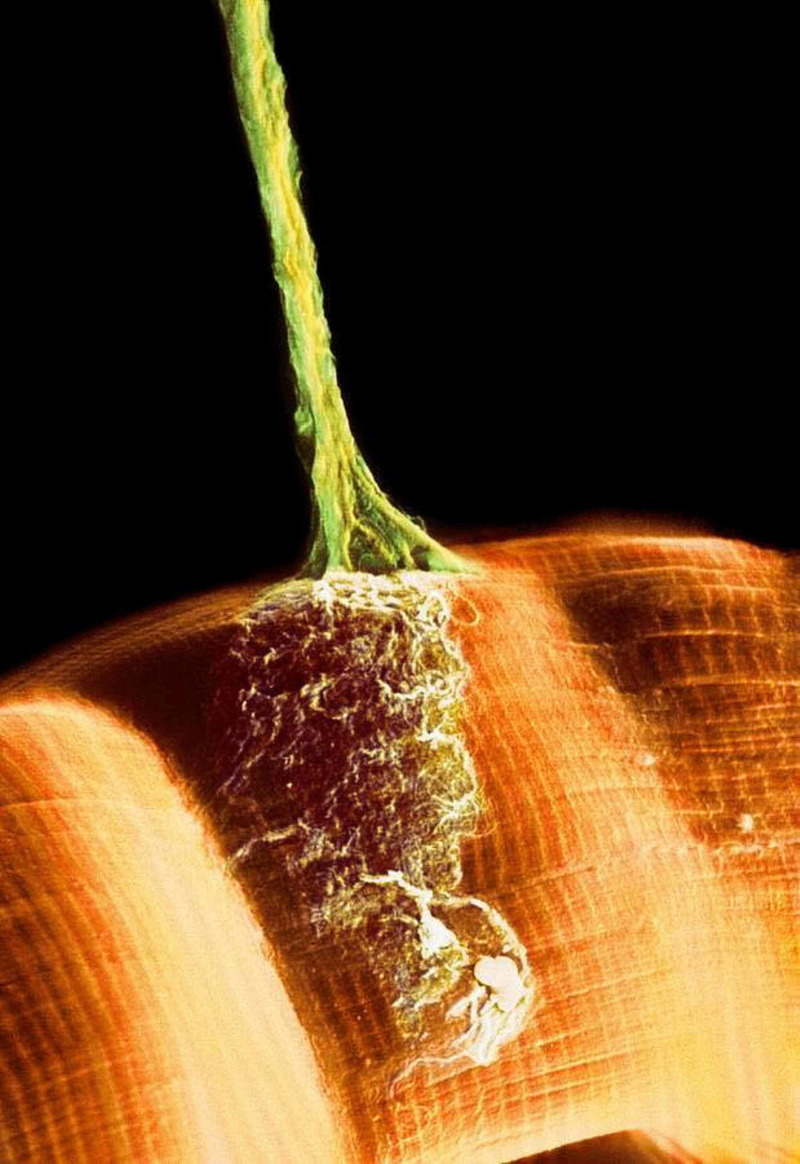
The figure illustrates an axon and synapses (in green) near muscle fibers (orange), by means of a colored micrograph under a scanning electron microscope (SEM). The image is taken from the Don Gnocchi chemistry and research laboratory in Milan.

The respiratory diaphragm is innervated by the phrenic nerve and the vagus nerve. The phrenic nerve arises from the spinal roots of C3-C5 (rarely involving C6), with phrenic neurons placed in the lamina IX of the ventral horn [28]. Phrenic neurons receive impulses from the medullary area of the preBötzinger complex and from neurons of the parafacial and retrotrapezoid complex; these three areas receive efferences from the retroambiguous nucleus of the bulb [28]. The preBötzinger area is a ventral portion of the medulla oblongata. The neurons that make up the latter area, for about 20% of the total, send autonomously (neural pacemaker) efferences to the phrenic neurons and to supramedullary areas, such as the hypothalamus, the amygdala, the thalamus, the cortex, and the area periaqueductal gray. The retrotrapezoid nucleus and the parafacial respiratory group are positioned rostrally to the preBötzinger group. Another area that stimulates the retrotrapezoid medullary area is the Kölliker-Fuse nucleus, in the portion of the pons, which nucleus receives efferences from the solitary nucleus (medulla oblongata) [29]. Kölliker-Fuse nucleus and medial parabrachial nuclei also send efferences to the preBötzinger complex. During the pre-inspiratory phase, there is evidence for activation of the pre-motor neurons linked to the hypoglossal and vagus nerve [28]. The proprioceptors of the muscles innervated by the upper cervical tract send afferents to the intermedius nucleus of the medulla, which sends afferents to the nucleus of the solitary tract; the latter will indirectly stimulate the retrotrapezoid medullary area [30]. The intermedius nucleus sends efferences to the nucleus of the XII cranial nerve [30]. The preBötzinger area not only sends efferences to the phrenic nerve, but to the XII nerve and trigeminal neurons, creating a symmetrical response of neural activity during the respiratory act [31]. The phrenic nerve crosses the prevertebral fascia and descends obliquely in front and above the anterior scalene muscle, covered by the fascia of the same muscle, posterior to the sternocleidomastoid muscle, touching the omohyoid muscle posteriorly; it descends anterior to the subclavian artery and posterior to the subclavian vein to enter the thorax [28]. At the cervical level, the phrenic nerve has several accessory nerves, which make anastomosis with the ansa cervicalis, the sympathetic cervical trunk (including the stellate ganglion), the sternohyoid nerve and the sternohyoid nerve, the vagus nerve and the hypoglossal nerve, the nerve accessory, the supraclavicular nerve, the subclavian nerve [28]. The phrenic nerve involves the cervical plexus and the BP, as well as some cranial nerves, continuing the neurological continuum between the tentorium cerebelli, the lingual complex, the thoracic outlet, and the respiratory diaphragm. The phrenic nerve can have accessory nerves and create a loop between the subclavian vein and the internal thoracic artery, or contribute to the cardiac plexus. When it passes the thoracic outlet and penetrates the chest, it comes into contact with the parietal pleura and with the visceral pericardium, which last covers the nerve. The right phrenic nerve is more vertical and shorter in length, and has faster electrical conduction than the left nerve; the right nerve has close relationships with the right atrium and the right mammary artery (from which a twig derives to vascularize the nerve), while the left nerve has relationships with the left ventricle and the mammary artery left [28]. The right phrenic nerve penetrates the connective area of the diaphragm muscle (phrenic center), the hiatus of the vena cava, from which it is wrapped by the fascia of the superior and inferior vena cava vessel; the left nerve penetrates the muscular area of the diaphragm [28]. Above and below the muscular portion of the diaphragm, the phrenic nerve can have additional accessory and collateral branches. When it crosses the diaphragm, the left nerve innervates the esophagogastric junction and the bottom of the stomach (apex of the bowel) with thin branches (gastric branch) [32]. The phrenic nerve with its phrenicoabdominal branches at subdiaphragmatic level innervates the parietal peritoneum and the peritoneal portion that surrounds the gallbladder, the Glisson capsule [28]. The right phrenic nerve, in particular, has several phrenic ganglia and to a lesser extent, the left nerve; accessory branches or phrenic post-ganglion branches are anastomosed to the celiac plexus/celiac ganglion, a complex known as phrenic plexus or celiac plexus branches [32]. The extension of the phrenic plexus involves the subdiaphragmatic surface, the suprarenal gland, the inferior vena cava, and the portion of the esophagus under the diaphragm and the sympathetic superior mesenteric ganglion. Branches directly from the phrenic ganglia can involve the suprarenal gland. The phrenic nerve sends efferences to the muscle tissues and receives afferents from the viscera; is able to send somatic, visceral, and nociceptive information. The phrenic nerve and in particular its ganglia have sympathetic components. Via the spinothalamic pathway and at the height of the medullary segment C2-C3, the phrenic afferents overlap the visceral and trigeminal vagal afferents, and precisely in the dorsal areas of the laminae I, II, III, and IV [33]. The vagus nerve or cranial X is located starting from the ambiguous nucleus, from the solitary nucleus, and from the dorsal motor nucleus of the brain stem, immediately caudal to the glossopharyngeal; the dorsal nucleus (or cardiopneumoenteric nucleus) which is found in the bulb under the floor of the fourth ventricle, gives rise to the parasympathetic preganglionic fibers of the vagus that come out of the brain stem [28]. The nerve in output from its nuclei moves horizontally forward and obliquely, to reach the jugular foramen; at this level, it crosses the bone canal forming two ganglia. The first ganglion, in the jugular foramen, is the superior with sensory tasks, while the second called inferior or nodose is below the jugular foramen (near the transverse process of C1) has somatic relevance; both ganglia are afferent. The vagus performs several anastomoses in the cervical portion, including the sympathetic system (including the stellate ganglion), the pharyngeal plexus, the phrenic nerve, the vagus nerve itself (Galen's anastomosis) via the Galenus nerve (between the posterior branches of the internal laryngeal nerve and the recurrent laryngeal nerve), nerve XI (Lobstein's anastomosis), nerve IX and with the ansa cervicalis and the spinal trigeminal nucleus [28]. The vagus nerve contains different types of fibers differentiated according to the speed of the electrical impulse and size. We can recognize myelinated fibers (A-fibers) capable of carrying efferent information (versus smooth muscle and pre-ganglionic fibers) and afferent information (proprioceptive, interoceptive, thermal, and nociceptive sensations); A-fibers with a smaller diameter and always myelinated for the afferent transport of visceral information [34]. The B-fibers send sympathetic information and parasympathetic pre-ganglionic terminations; we find small nonmyelinated C-fibers for visceral afferents [34]. The efferent fibers derive mainly from the nucleus ambiguous and from the dorsal motor nucleus; the vagal afferent fibers travel to the postrema area, spinal trigeminal nucleus, and to the nucleus of the solitary tract [34]. The vagus joins the carotid (carotid sheath) and the jugular vein, piercing the deep fascia of the neck; it splits in two, forming the recurrent laryngeal nerves, which will create loops around the aortic arch (left) and around the right subclavian artery. The superior laryngeal nerve is a branch of the vagus nerve that arises after the passage from the larynx, which branch anastomizes with a post-ganglion branch of the upper cervical sympathetic ganglion [34]. Entering the mediastinum, the X nerve contacts all the viscera, as well as the viscera of the abdomen and pelvis. At the diaphragmatic level, the vagus nerve innervates the muscular portion of the esophagal hiatus, the phrenic or Lamier ligaments [33]. In the cervical tract, a nonrecurrent laryngeal nerve can be found with very low percentages, which enters the larynx with some thyroid arteries. At the subdiaphragmatic level, the vagus nerve will create anastomosis with the sympathetic ganglia of the abdomen, phrenic plexus, and phrenic nerve. The nervous continuum connects the tentorium cerebelli, the lingual complex, the thoracic outlet, and the respiratory diaphragm (Figure 2).

Pelvic floor

The pelvic floor is a large but thin muscle layer that forms the lower edge of the abdominopelvic cavity and between the pubic symphysis and the coccyx; the musculature is formed by the levator ani (puborectal, pubococcygeus, and iliococcygeus muscles) and the ischiococcygeus muscle [2]. During inspiration, the medullary and supramedullary breathing centers activate the XII cranial nerve to retrude the tongue, while the respiratory diaphragm is lowered, the musculature of the abdomen contracts (to prevent excessive forward movement of the diaphragm), the musculature deep back has less electrical activity, and the muscles of the pelvic floor are released (drops) [28]. The pelvic floor innervation derives from upper centers and neurons from the nucleus of Onuf (lamina IX of the sacral spinal cord from the anterior horns) and from the pudendal nerve (whose motor neurons derived from the same medullary level) or S2-S4 [35]. The pudendal nerve, motor and sensory, exits the pelvis for the lower part of the greater ischiatic foramen, between the piriformis muscle and the ischio-coccygeal muscle, enters the gluteal region by crossing the sacrospinous ligament, passes through the lesser ischiatic foramen where it is wrapped by a fascia stretched between the lower portion of the obturator fascia and the sacrotuberous ligament, forming the pudendal canal (Alcock canal) [35]. In its posterior tract, it emits the lower rectal nerve (also called hemorrhoidal); proceeding anteriorly it divides into the perineum nerve and the dorsal nerve of the penis (or clitoris). The inferior rectal nerve emits several branches, which anastomize with the perineal branch of the posterior cutaneous nerve of the thigh and with the scrotal (or labial) nerves of the perineum nerve; the nerve of the perineum, the lower terminal branch is divided into posterior scrotal (or labial) branches and muscular branches [35]. The cell bodies of the pre-ganglion neurons of the sacral parasympathetic are located in the intermediate-lateral part of the S2-S4 segments of the spinal cord. The axons of the pre-ganglionic neurons leave the medulla through the anterior roots, run into the pelvic nerves, and reach the pelvic plexus ganglia whose neurons are located near and within the wall of the target organs; at the anal canal level, the parasympathetic post-ganglionic fibers synapse with cholinergic myenteric neurons that innervate the longitudinal muscle and nonadrenergic noncholinergic neurons that innervate the circular muscle of the internal sphincter of the anus [36]. Pre-ganglionic cells of sympathetic derivation form a column that extends from the first thoracic segment to the first lumbar segments. The cell bodies are arranged at the level of the intermediate-lateral gray substance; the preganglionic axons leave the spinal cord with the ventral roots and become part of the spinal nerve, then abandon the somatic fibers and form the white communicating branches that lead to the ganglia of the paravertebral chain [36]. The post-ganglionic fibers that originate from neurons of the ganglia of the sympathetic trunk, thin and myelinated, are distributed to the effector organs directly following a peripheral branch of the ganglion to reach a viscera or some other territory, or they can pass through a gray communicating branch to the spinal nerve. The gray communicating branches can reach their spinal nerve either directly from the corresponding ganglia or by ascending or descending into the sympathetic trunk to reach the spinal nerves of the cranial and sacral segments [36]. The sympathetic nerves that derive from the trunk (greater splanchnic nerve, T5-T10; lesser splanchnic nerve, T9-T12; least splanchnic nerve or renal nerve, T12) and which pass through the diaphragm muscle, will form the celiac ganglion, the upper mesenteric ganglion, and the renal aortic ganglion; these three ganglia will meet sympathetic fibers from the lumbar sympathetic plexus and will form a continuum with the upper and lower hypogastric plexus (after the aortic bifurcation) [37]. The renal aortic ganglion contains sympathetic and parasympathetic fibers and joins the hypogastric plexus (L4-L5); the sympathetic sacral fibers derive from T12-L2, while the parasympathetic fibers that will form anastomosis with sympathetic fibers derive from S2-S4. The pudendal nerve has autonomic components and is activated with the phrenic nerve by the actions of the pelvic floor, not simply combined with breathing (walking, posture) [28, 38]. From the neurological point of view, we can say that there is a continuum between the five diaphragms, just as there is a myofascial continuum [1-2].

Clinical reflections

**Figure 3:**
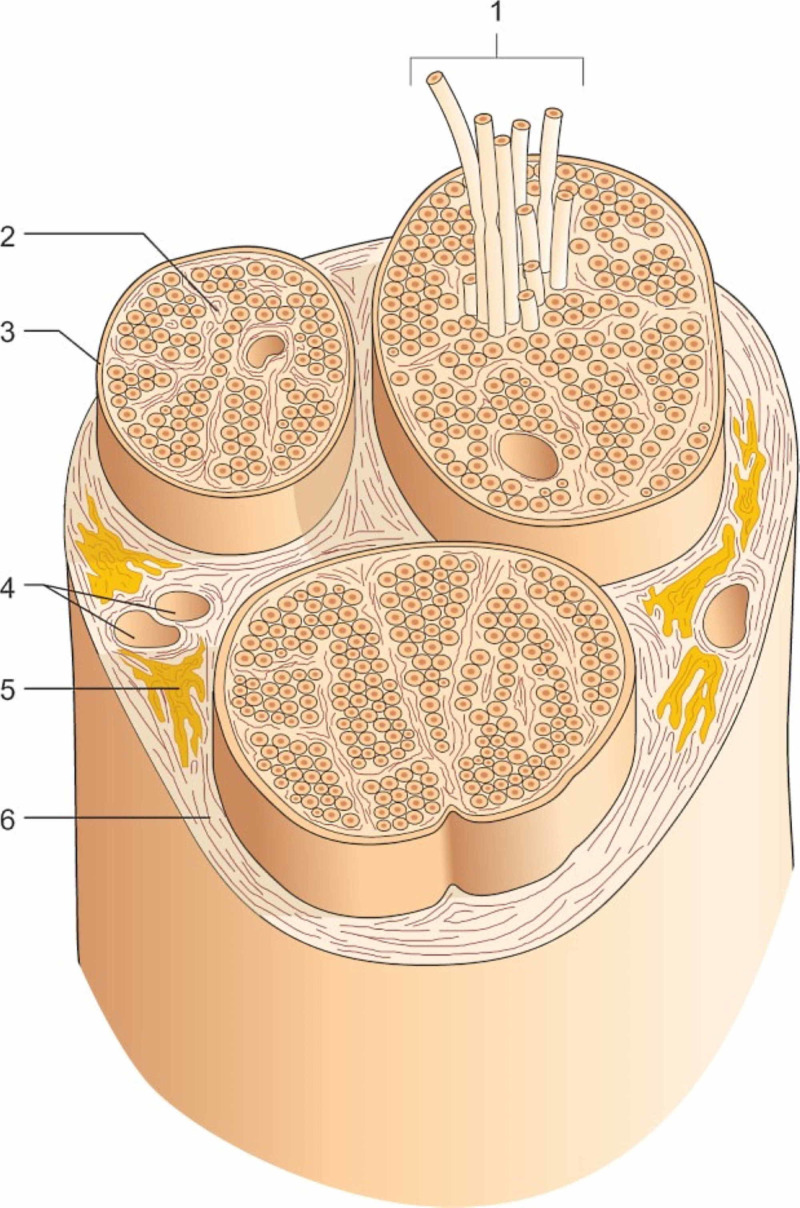
Constitution of a nerve in transverse section in a classic view of the nerve. 1: Nerve bundles; 2: The endoneurium; 3: The perineurium; 4: Blood vessels; 5: Fat; 6: The epineurium. Reproduced with permission, from Anastasi G, et al., Anatomia dell'uomo, fourth edition [Human Anatomy], 2010, Milan: Edi-Ermes, Volume 3, p. 232.

The nerve is like a highway, with multiple lanes and two-way traffic; it is crossed in its path by fast or slow vehicles of all shapes. The toll stations may be the ganglia, interneurons, and the different receptors or the multiple synaptic relationships. The nerve is made up of layers and we can distinguish an outermost layer or epineurium, perineurium, and endoneurium. The epineurium, in its internal portion, contains vessels and adipose tissue and envelops the branches of the nerve; the endoneurium is a connective tissue sheath, which has the task of covering the axon, while the perineurium covers and holds together several axon bundles. Perineurium together with endoneurium constitutes a blood-nerve barrier [39]. The number of files varies from 1 to 200, depending on the size of the nerve; the arrangement of the bundles may also vary according to the function of the nerve. According to recent data, there is a further layer of connective tissue in indirect contact with the perineurium, called internal epineurium, which last is in contact with the first layer but without adipose tissue between the two layers; the internal epineurium covers two or more files [39]. To put the nerve in direct contact with the adventitia of the vessels and the epimysium of the muscles is a layer called circumneurium or paraneurium [39]. The nerve is defined on the basis of its function and characteristic (sensory, motor); in reality, it does not take into account the fact that the nerve not only carries electricity (Figure 3).

**Figure 4:**
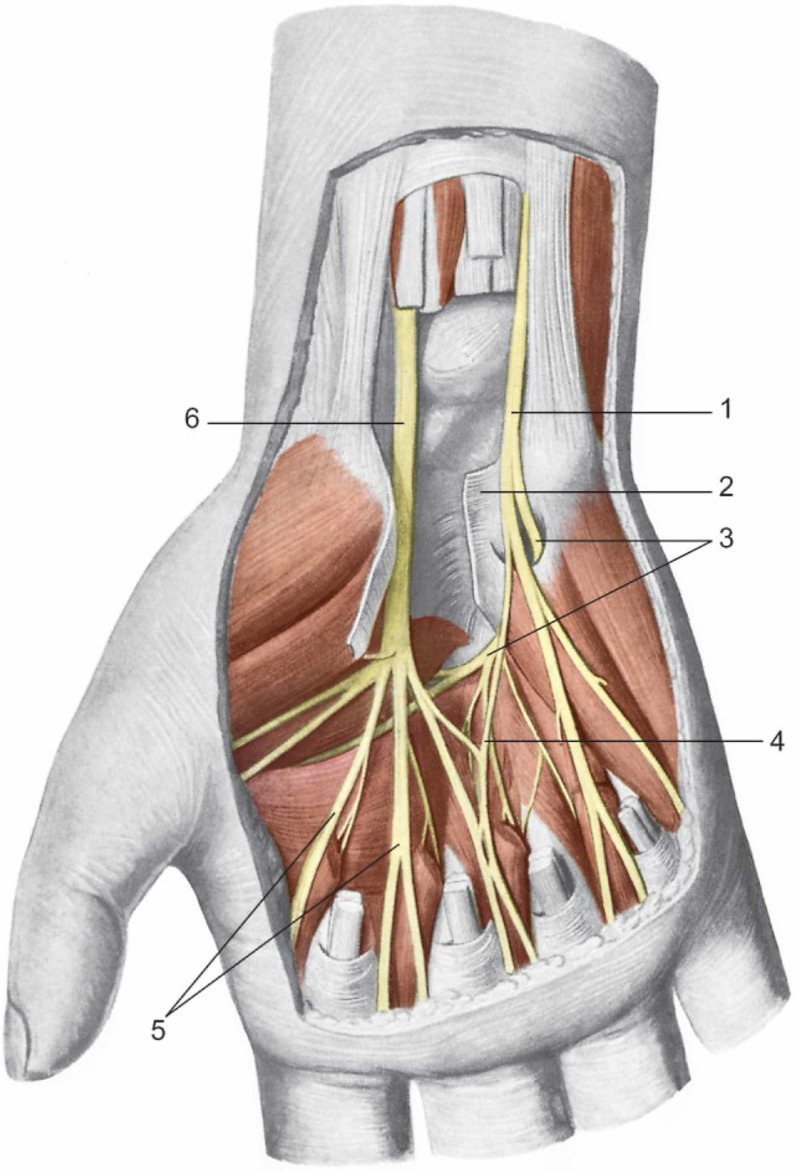
Distribution of the median and ulnar nerves in the palmar region of the hand. The two nerves reach the palm region with their palm branches and contract numerous anastomoses. They divide forming digital nerves. The median nerve runs deeply to the transverse ligament of the carpus, while the ulnar nerve passes superficially to it. 1: The ulnar nerve; 2: The transverse carpal ligament; 3: Deep palmar branch of the hand; 4: Anastomosis between the median nerve and ulnar nerve; 5: Digital nerves; 6: The median nerve. Reproduced with permission, from Anastasi G, et al., Anatomia dell'uomo, fourth edition [Human Anatomy], 2010, Milan: Edi-Ermes, Volume 3, p 260.

If we consider the ability of the nervous tissue to transport multiple substances in a biunivocal direction and that these substances (growth factors, immune substances, hormones) can change not only the target tissue (muscle and spinal cord, the structure and phenotype of the motor neuron) but, even the same behaviour of the nerve (from efferent to afferent), it becomes evident that the mere division into the somatic, sensory, or mixed nerve is reductive [28]. To give an example, if the capacity of the respiratory diaphragm muscle and its individual fibers have a physiological space in which to express their contraction, it will produce a greater amount of growth factors including the brain-derived neurotrophic factor (BDNF) [40]. Retrograde BDNF will change the properties of the peripheral synaptic plate, improving its ability to release acetylcholine, and will change the morphology of the synaptic plate to make it more compliant to muscle fiber [40]. Depending on muscle contraction (under aerobic or anerobic stimuli), the quantity and type of neurotrophic factors synthesized by muscle fibers change. With a retrograde path on the efferent axon, BDNF can contact the motor neuron, changing its morphology and phenotype [28]. The retrograde transport of multiple substances is a tool that the nervous system uses to maintain the development and maintenance of the health of the nervous complex [41]. These substances, including neurotrophins (BDNF, nerve growth factor or NGF) are produced post-synaptically and transported retrogradely through endosomes. Neurotrophins bind to specific receptors (NGF binds to tyrosine kinase (Trk) receptor; BDNF binds to Trk type B and C), constituting a complex structure that in cascade can stimulate the formation of new dendrites, change the shape-function of the neural body, to allow synaptogenesis [41]. The change produced by the stimulation of muscle contraction to the peripheral and central nervous system will be temporary or definitive. Constant mechanical stimuli from the musculoskeletal system will condition the nervous system in its form and function [42]. The bi-directionality of transmitting different information from the nerve towards the tissues, towards other nerves and towards the medulla or higher structures, allows understanding many clinical aspects. Studies show that in the carpal tunnel syndrome the median nerve is in pain. In this context, there is a stimulation of the sympathetic system, altering the behavior of the sweat glands; the skin temperature decreases not because of vasoconstriction of the vessels, but rather because of an increase in the production of the sweat glands [43]. The altered mechanical behavior of the tissues penetrated by the nerves causes an alteration of the median nerve, the BP, and the sympathetic system (Figure 4) [43].

It is not possible to think that if a nerve is suffering in a specific area (compression, traction, ischemia) the remaining collateral nerves, the entire path of the nerve and plexus (in this example the BP) are free from disturbances (sub-clinical) [44-45]. A striking example of what has been said comes from animal studies with stimulation of the median nerve. Median nerve stimulation has been shown to have a systemic anti-sympathetic effect, stimulating the cholinergic anti-inflammatory pathway response; probably, this happens for retrograde stimulation of the spinal nerve up to the cervical parasympathetic system [46]. The vagus nerve enhances its response with a decrease in the production of pro-inflammatory cytokines (tumor necrosis factor alpha or TNFα, interleukin-6 or IL-6) [46]. A suffering median nerve, as in the case of carpal tunnel syndrome, will have a systemic opposite effect, that is, a systemic increase in inflammatory substances with a sub-clinical inflammatory picture [47]. Taking another example to understand anatomical continuity is the appearance of migraine headaches, involving the tentorium cerebelli and the respiratory diaphragm. If the sub-occipital musculature is in a state of constant contraction, with lack of space to stretch and shorten properly, they can stimulate head pain. The movement deficit of these muscles will cause the production of inflammatory substances from the contractile tissue, which substances will travel inside the skull, through the spinal nerves that innervate the sub-tentorial area [48]. Retrogradely the C1-C2 nerves will carry a multitude of pro-inflammatory substances and some specific proteins in the skull. Spinal effector axons are positive for calcitonin gene-related peptide (CGRP) and for transient receptor potential cation channel subfamily V member 1 or TrpV1, proteins involved in nociception [48]. These proteins can stimulate vasodilation and sub-tentorial dural inflammation (mast cells and macrophages) which will stimulate a peripheral cholinergic response and trigeminal meningeal stimulation and pain. Parasympathetic overexcitation can increase cholinergic tone in the bronchi and cause asthma; the presence of asthma will cause a series of biochemical responses transported retrogradely from the vagus nerve to the tentorium, stimulating further inflammation and pain in the head, in a vicious circle [49]. Pain and inflammation will stimulate the activity of the transient receptor potential cation channel, subfamily member 1 or TRPA1, which is located on the neural tissue, which in cascade will stimulate the production of acetylcholine (ACh) and CGRP. The mast cells themselves can travel bi-directionally from the spinal nerve to the dura mater and from the dura mater to the nerves involving the sub-occipital muscles [50]. A simple contracture of sub-occipital muscles, through the nervous system, can trigger a symptomatic picture that is not always easy to classify. The space between the tissues generates health and movement [5]. A dysfunction of a specific area with limited space of movement (sliding, stretching, and contraction) can create malfunctions to the whole system [28]. Trying to deal with a clinical picture, not only starting from the symptom but, with a global bodily perspective that takes into consideration the anatomical, neurological and myofascial continuum as in the OMM approach of the five diaphragms, should allow for a better clinical interpretation. We find the anatomical continuum perfectly in the nervous complex, as highlighted in the article divided into two parts. The nerve layers are the continuation of the cranial meninges: the perinerium is the continuity of the arachnoid tissue; epineurium is the continuation of the dura mater; the endoneurium is the continuation of the pia mater [5].

## Conclusions

In this second and final part of the article the neurological continuum has been illustrated, which connects the five diaphragms, body structures which, in addition to the respiratory diaphragm, can be considered as diaphragms in OMM: tentorium cerebelli, tongue, thoracic outlet, respiratory diaphragm, and pelvic floor. The nervous system is in constant communication, not only through electrical activity but also with different biochemical structures that are able to travel through the nerve/nerves in a bi-directional way. This allows communication of the nervous tissue and extra-nervous tissues. Patient health begins with the possibility of movement of the different tissues (space), which is maintained by a systemic physiological movement. Recall that the nerve also has the function of detoxifying the tissues, which are innervated and perforated by the nerve itself, thanks to the ability to move multiple molecules in its layers; this happens if the space allows the correct movement. The beginning is the boundary of entropy; senescence is the negentropic centrosome: the beginning of the movement leads to the ability of a better adaptation in the different planes and axes of the tissue/cell, while immobility leads to the death of the tissue/cell with the inability to adapt, starting from the demolition of the nucleus itself.
